# Association between 20q12 rs13041247 polymorphism and risk of nonsyndromic cleft lip with or without cleft palate: a meta-analysis

**DOI:** 10.1186/s12903-020-1003-2

**Published:** 2020-02-04

**Authors:** Liheng Huang, Xinglong Liang, Yangzhan Ou, Shijie Tang, Yunpu He

**Affiliations:** 1 Department of Anesthesiology, Shantou Central Hospital, Affiliated Shantou Hospital of Sun Yat-sen University, Shantou, Guangdong China; 2 Department of Dermatology, Maoming People’s Hospital, Maoming, Guangdong China; 3 Department of Plastic Surgery and Burn Center, the Second Affiliated Hospital, Shantou University Medical College, No.69 North Dong Xia Road, Shantou, 515041 Guangdong China

**Keywords:** Meta-analysis, Nonsyndromic cleft lip with or without cleft palate, MAFB, 20q12, rs13041247

## Abstract

**Background:**

Previous genome-wide association studies have identified a link between the rs13041247 single nucleotide polymorphisms (SNPs) in the chromosome 20q12 locus and the development of the congenital malformation known as nonsyndromic cleft lip with or without cleft palate (NSCL/P). The present meta-analysis was therefore designed to formally assess the relationship between rs13041247 and NSCL/P.

**Methods:**

We searched Embase, Web of Science, PubMed, the China National Knowledge Internet (CNKI), and the China Wanfang database in order to identify relevant published through 25 June 2019. This allowed us to identify 13 studies incorporating 4914 patients and 5981 controls for whom rs13041247 genotyping had been conducted, with STATA 12.0 then being used to conduct a meta-analysis of these pooled results. The I^2^ statistic was used to compare heterogeneity among studies.

**Results:**

In total this analysis incorporated 13 case-control studies. No association between the rs13041247 polymorphism and NSCL/P risk was detected in individuals of Asian ethnicity (C vs T: OR = 0.847, 95% CI = 0.702–1.021; CC vs TT: OR = 0.725, 95% CI = 0.494–1.063; CC vs CT: OR = 0.837, 95% CI = 0.657–1.067; CT + TT vs CC: OR = 1.265, 95% CI = 0.951–1.684; CC + CT vs TT: OR = 0.805, 95% CI = 0.630–1.029) or Caucasian ethnicity (C vs T: OR = 0.936, 95% CI = 0.786–1.114; CC vs TT: OR = 0.988, 95% CI = 0.674–1.446; CC vs CT: OR = 1.197, 95% CI = 0.816–1.757; CT + TT vs CC: OR = 0.918, 95% CI = 0.639–1.318; CC + CT vs TT: OR = 0.855, 95% CI = 0.677–1.081). However, an overall analysis of all participants in these studies revealed the rs13041247 C allele, the CT genotype, and the CC + CT model to be linked to a reduced NSCL/P risk (C vs T: OR = 0.897, 95% CI: 0.723–1.114, *P* = 0.048; CT vs TT: OR = 0.839, 95% CI: 0.734–0.959, *P* = 0.01; CC + CT vs TT: OR = 0.824, 95% CI: 0.701–0.968, *P* = 0.019).

**Conclusion:**

These results suggest that the rs13041247 SNP located at the 20q12 chromosomal locus is associated with NSCL/P risk in an overall pooled study population, although this association was not significant in East Asian or Caucasian populations.

## Background

Non-syndromic cleft lip with or without cleft palate (NSCL/P) is a congenital birth defect that affects a relatively high percentage of individuals in a manner that is linked to ethnicity [1], with rates ranging from 1.423/1000 in Chinese populations [2] to 1/500 in American Indian and Asian populations [3].

Both environmental and genetic factors can regulate the development of NSCL/P, but the underlying mechanisms are not fully understood at present. Recent genome-wide association studies have revealed that polymorphisms in the genomic region encoding the v-maf musculoaponeurotic fibrosarcoma oncogene homolog B (MAFB) are strongly associated with the risk of NSCL/P [4]. In line with this finding, multiple NSCL/P-related variants that may be causative of this condition have been identified in the 20q11.2 region of the genome, which is proximal to the MAFB gene [5].

Research regarding the rs13041247 polymorphism within the 20q12 chromosomal locus has provided strong evidence for the relevance of this single nucleotide polymorphism (SNP) to NSCL/P incidence Indian, Brazilian, Mesoamerican, and Chinese populations [5–8]. However, results have been inconsistent in different study populations, potentially due to the differences in ethnicity between these patient cohorts.

In order to fully explore the role of the rs1304127 in NSCL/P, the present meta-analysis of previously published case-control studies was conducted. This approach was employed in order to attain additional statistical power, allowing for the thorough examination of the relationship between this SNP and this congenital malformation in different populations. The results of this study have the potential to guide genetic counseling efforts in families at risk of or affected by NSCL/P.

## Methods

### Articles selection

Embase, Web of Science, PubMed, the China National Knowledge Internet (CNKI) and the China Wanfang database were searched for articles published through 25 June 2019. Search terms used were as follows: (“nonsyndromic cleft lip with or without cleft palate” or “cleft palate” or “cleft lip” or “orofacial clefts” or “oral cleft” or “CL” or “CP” or “NSCL/P”) and (“MAFB” or “20q12” or “v-maf musculoaponeurotic fibrosarcoma oncogene homolog B”) and (“polymorphism” or “allele” or “gene” or “SNP”). No restrictions were imposed upon the language in which articles were published.

### Study selection and data extraction

In order to be included in this meta-analysis, studies had to meet the following inclusion criteria: (1) Studies were case-control studies; (2) Studies were focused on NSCL/P; (3) Polymorphisms analyzed in the study included rs13041247 at the 20q12 locus; (4) Studies provided sufficient data necessary for the calculation of odds ratios (ORs) and 95% confidence intervals (CIs); (5) All necessary data was either available or was obtained within two attempts to contact the study authors. When multiple articles included overlapping patient cohorts, only the study with the most comprehensive information was included in the present meta-analysis (i.e. the study with the largest population or the most complete dataset). In addition, studies meeting the following criteria were excluded from this analysis: (1) Animals studies; (2) Reviews, letters, or abstracts lacking original data; (3) Case-reports; (4) Studies focused only on other SNPs or which lacked a control group. No language restrictions were imposed on these studies.

Two authors (Liheng Huang and Yangzhan Ou), independently reviewed identified studies in order to exclude those which were either clearly irrelevant or which were duplicates, after which a full text review was performed to identify studies meeting the inclusion criteria for this analysis. Any discrepancies were resolved via discussion with the third author (Yunpu He).The following pieces of data were extracted from these studies: Name of the first author, year of publication, country, population ethnicities, control source, study design, samples size, control Hardy-Weinberg equilibrium (HWE) *p*-values, genotyping methodology, and case/control genotype distributions (Table 1) [5–17]. In addition, the Newcastle-Ottawa scale was used by two authors (Yunpu He and Shijie Tang) to independently evaluate included study quality as recommended previously [18]. In total, 8 of the included studies reported on polymorphisms in Chinese and East Asian populations, 2 focused on Caucasian populations Germany and Brazil [15, 16], and 1 each focused on Nigerian [17], Brazilian [7], Mayan Mesoamerican [8], Indian [5] ethnic populations. For those subgroups for which more than one article was available, ethnicity-based subgroup analyses were performed.

**Table 1:** The basic information and data of 13 studies in the meta-analysis

First author/Year	Country	Ethnicity	Study design	Source of	Genotyping	Study size	P for HWE	Case	Control	Case	Control
				Controls	method	case/control	in controls	CC/CT/TT	CC/CT/TT	C/T	C/T
Enmin Huang 2012 [9]	China	East Asian	Case-control	PB	Mass spectrometry	300/354	0.689	33/149/118	70/179/105	215/385	319/389
Lang Feifei 2017 [10]	China	East Asian	Case-control	PB	TaqMan	162/178	> 0.05	43/69/50	22/85/71	155/169	129/227
Xiaoqing Yin 2018 [6]	China	East Asian	Case-control	PB	TaqMan	1278/1295	0.331	202/606/458	270/640/374	1010/1522	1180/1388
Zhongwei Z 2013 [11]	China	East Asian	Case-control	PB	PCR-RFLP	369/433	0.99	62/162/49	145/211/77	286/260	501/365
Pan 2011 [12]	China	East Asian	Case-control	HB	PCR	367/382	0.26	50/159/158	89/202/91	259/475	380/384
Mi 2014 [13]	China	East Asian	Case-control	HB	Mini-sequencing	324/343	0.956	78/162/84	63/168/112	318/330	294/392
Sun 2015 (I) [14]	China	East Asian	Case-control	PB	Affymetrix Genome-Wide	504/455	0.991	77/250/203	110/258/151	404/656	478/560
Sun 2015(II) [14]	China	East Asian	Case-control	PB	Affymetrix Genome-Wide	384/793	0.977	74/189/121	185/399/216	337/431	769/831
Gurramkond 2015 [5]	Indian	India’s	Case-control	HB	KASPar	173/176	0.85	4/65/75	20/77/79	73/215	117/235
Reiter 2015 [15]	Germany	Caucasian	Case-control	PB	PCR	119/383	0.843	20/46/53	46/176/161	86/152	268/498
Clarissa Fontoura 2012 [16]	Brazil	Caucasian	Case-control	HB	TaqMan	400/412	0.51	38/165/182	42/180/166	241/529	264/512
Kerstin U.Ludwig 2014 [8]	Mexico	Mayan	Case-control	PB	PCR-RFLP	153/337	0.192	15/67/66	51/139/129	97/199	241/397
Do Rego Borges 2015 [7]	Brazil	Brazilian	Case-control	PB	PCR	293/352	0.964	29/100/164	25/137/190	158/428	187/517
Butali 2011 [17]	Nigerian	African	Case-control	PB	PCR	88/88	0.88	54/30/4	47/35/6	138/38	129/47

Abbreviations: HWE, Hardy-Weinberg equiibrium; PCR: polymerase chain reaction; RFLP: restriction fragment length polymorphism; KASPar, allele-specific amplification followed by fluorescence detection; PB, population-based; HB, hospital-based; (I) Huaxi Cohort; (II) Nanjing Cohort

### Statistical analysis

STATA 12.0 was used to obtain crude ORs and 95% CIs for each of the included articles as a means of assessing the relationship between the rs13041247 SNP and NSCL/P risk. Chi-squared tests were used to assess the HWE in the control group, revealing it not to differ significantly from HWE (*P* > 0.05). Ethnicity-based subgroup analyses were also conducted. Five different genetic models were used to examine the rs13041247 and NSCL/P risk according to ORs and 95% CIs: an allele model (C vs T), a homozygote model (CC vs TT), a heterozygote model (CC vs CT), a dominant model (CT + TT vs CC), and a recessive model (CC + CT vs TT). Z-tests were used to assess the significance of pooled ORs, *P* < 0.05 as the significance threshold. The I^2^ statistic was used to assess heterogeneity among studies, with I^2^ > 50% being consistent with significant heterogeneity, leading to the use of a random effect model, and I^2^ < 50% leading to the use of a fixed effect model. Sensitivity analyses were conducted by iteratively omitting individual studies from the overall analysis, while funnel plots were used to examine the risk of publication bias.

## Results

### Study characteristics

**Fig. 1:**
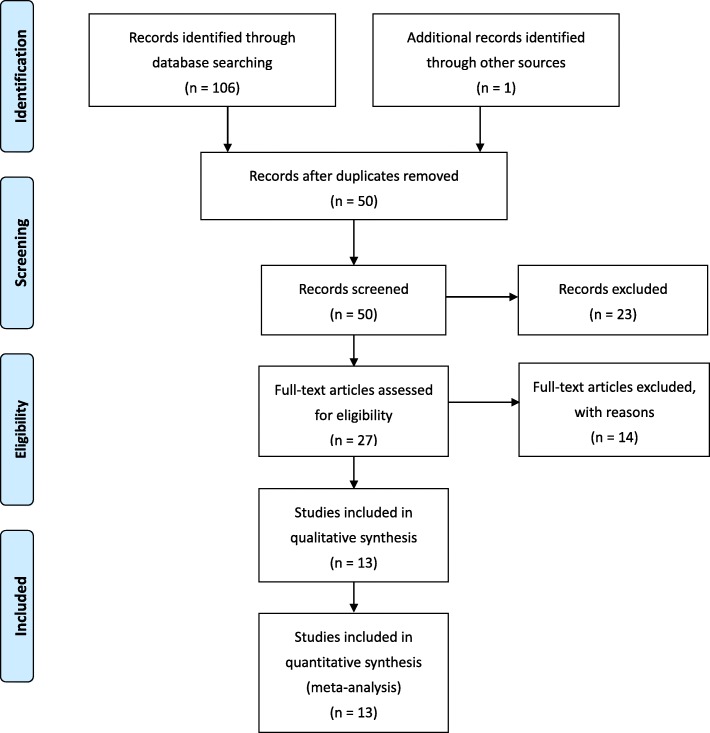
Flow diagram of study selection process

The study selection process for the present meta-analysis is detailed in Fig. 1, with the characteristics of included studies shown in Table 1. In total, 13 case-control studies were included in the present meta-analysis, incorporating a total of 4914 cases and 5981 controls in whom rs13041247 genotyping had been performed [5–17]. These studies included diverse populations from a range of ethnicities, including East Asian, Caucasian, India, Mayan, Brazilian, and African cohorts. The quality of these studies was assessed using the Newcastle-Ottawa scale (NOS), revealing all of these studies to be of high quality with scores > 7 out of a possible 9 (Table 2).

**Table 2:** Quality assessment scores for the studies included in this meta-analysis

First Author (year)	Selection	Comparability	Exposure	Total Points
Enmin Huang 2012 [9]	****	*	***	8
Lang Feifei 2017 [10]	***	*	***	7
Xiaoqing Yin 2018 [6]	****	**	***	9
ZHOU Zhongwei 2013 [11]	***	*	***	7
Pan 2011 [12]	***	**	***	8
Mi 2014 [13]	***	**	***	8
Sun 2015 (I) (II) [14]	****	*	***	8
Gurramkond 2015 [5]	***	**	***	8
Reiter 2015 [15]	***	*	***	7
Clarissa Fontoura 2012 [16]	***	*	***	7
Kerstin U.Ludwig 2014 [8]	****	–	***	7
Do Rego Borges 2015 [7]	****	–	***	7
Butali 2011 [17]	***	*	***	7

Each asterisk means one point

### Meta-analysis results

**Fig. 2:**
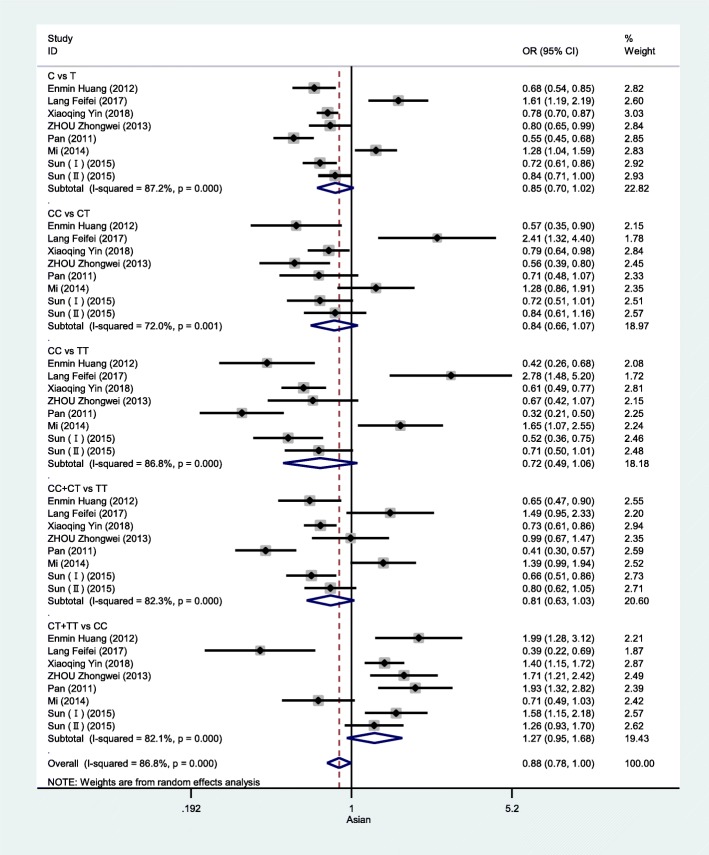
Forest plot of the association between rs13041247 and NSCL/P risk in East Asian ethnicity

**Fig. 3:**
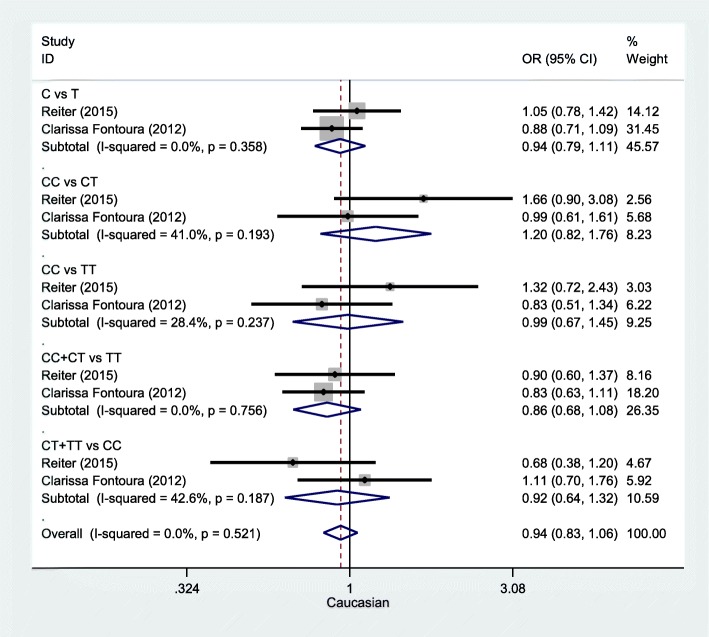
Forest plot of the association between rs13041247 and NSCL/P risk in Caucasian ethnicity

**Fig. 4:**
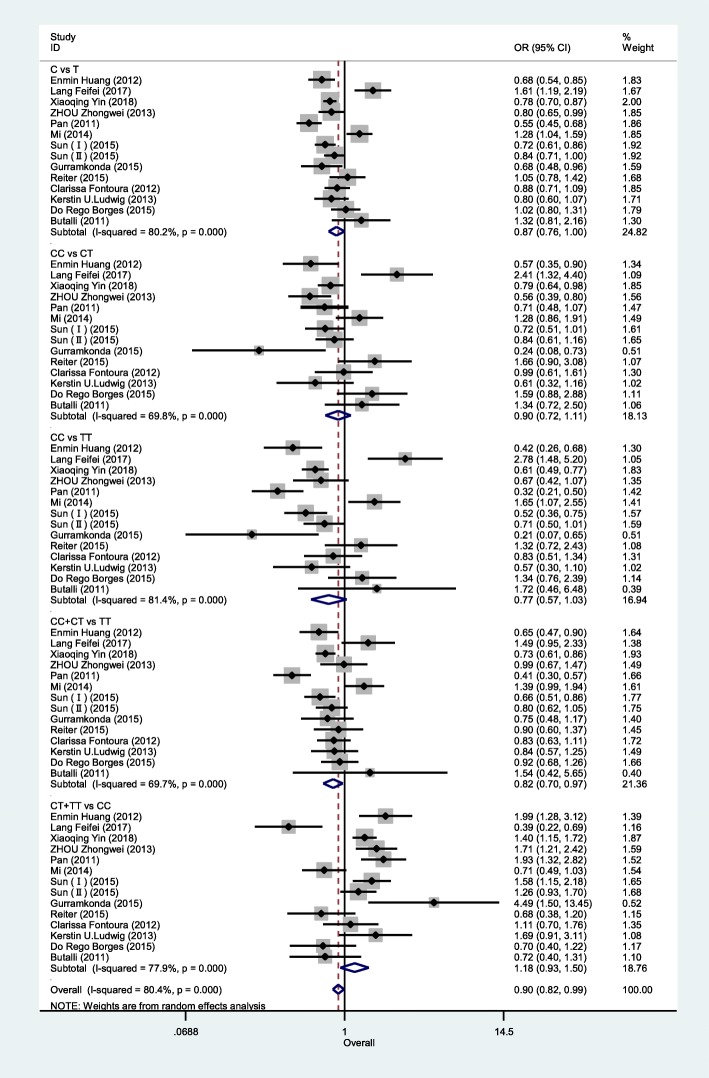
Forest plot of the association between rs13041247 and NSCL/P risk in Overall population

**Fig. 5:**
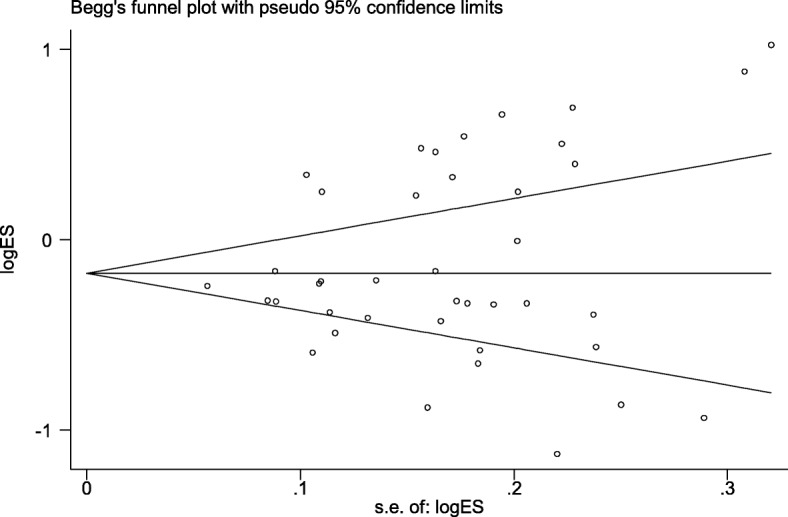
Funnel plot of the association between rs13041247 and NSCL/P risk in East Asian ethnicity

**Fig. 6:**
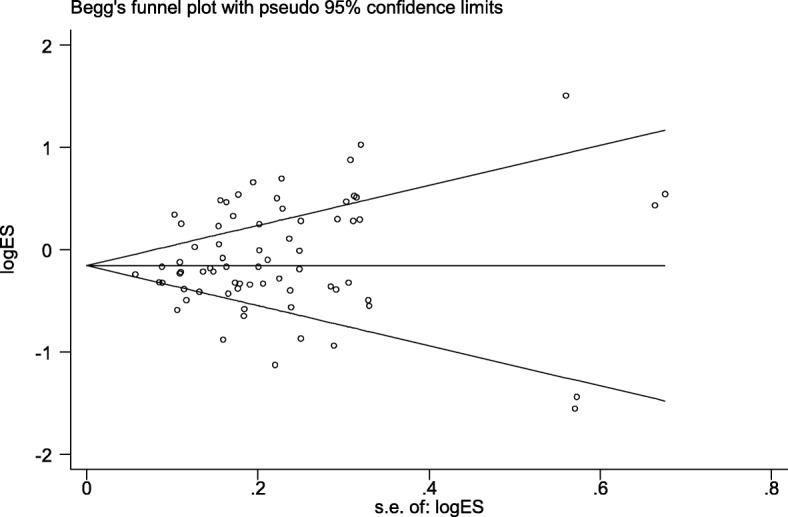
Funnel plot of the association between rs13041247 and NSCL/P risk in Overall population

Table 3 highlights the results of this meta-analysis of the relationship between rs13041247 and NSCL/P risk. Ultimately, this analysis revealed no significant association between rs13041247 and NSCL/P risk in an East Asian population (C vs T: OR = 0.847, 95% CI = 0.702–1.021; CC vs TT: OR = 0.725, 95% CI = 0.494–1.063; CC vs CT: OR = 0.837, 95% CI = 0.657–1.067; CT + TT vs CC: OR = 1.265, 95% CI = 0.951–1.684; CC + CT vs TT: OR = 0.805, 95% CI = 0.630–1.029) or in a Caucasian population (C vs T: OR = 0.936, 95% CI = 0.786–1.114; CC vs TT: OR = 0.988, 95% CI = 0.674–1.446; CC vs CT: OR = 1.197, 95% CI = 0.816–1.757; CT + TT vs CC: OR = 0.918, 95% CI = 0.639–1.318; CC + CT vs TT: OR = 0.855, 95% CI = 0.677–1.081). However, when the overall combined patient/control populations were analyzed, this analysis revealed that the C allele, the CT genotype, and the CC + CT model to be associated with significantly reduced NSCL/P risk (C vs T: OR = 0.897, 95% CI: 0.723–1.114, *P* = 0.048; CT vs TT: OR = 0.839, 95% CI: 0.734–0.959, *P* = 0.01; CC + CT vs TT: OR = 0.824, 95% CI: 0.701–0.968, *P* = 0.019). Significantly heterogeneity was detected in all models for both the overall and Asian analysis groups (I^2^ > 50%), whereas the heterogeneity in the Caucasian subgroup analysis was not significant. The results of this meta-analysis of the relationship between rs13041247 and NSCL/P risk for the allele model (C vs T), the heterozygote model (CC vs CT), the homozygote model (CC vs TT), the recessive model (CC + CT vs TT), and the dominant model (CT + TT vs CC) are shown in Figs. 2, 3, and 4. No significant changes in the study outcomes were detected in a sensitivity analysis, and no evidence of publication bias was detected based upon Egger’s test (East Asian *P* = 0.253, Caucasian *P* = 0.239, and Overall population *P* = 0.124). Similarly, no funnel plot asymmetry was detected (Figs. 5 and 6 for East Asian and Overall, respectively).

**Table 3:** Summary results of the association between polymorphism and NSCL/P risk in the meta-analysis

Subgroup	Ethnicity	Genotype	No of studies	Test of association				Test of heterogeneity	
				OR(95%CI)	Z-test	P-value	Model	P-value	I^2^ (%)
rs13041247	East Asian	C vs T	8	0.847 (0.702–1.021)	1.74	0.082	R	0.000	87.2%
		CC vs CT	8	0.837 (0.657–1.067)	1.43	0.151	R	0.001	72%
		CC vs TT	8	0.725 (0.494–1.063)	1.65	0.100	R	0.000	86.8%
		CC + CT vs TT	8	0.805 (0.630–1.029)	1.73	0.083	R	0.000	82.3%
		CT + TT vs CC	8	1.265 (0.951–1.684)	1.61	0.106	R	0.000	82.1%
rs13041247	Caucasian	C vs T	2	0.936 (0.786–1.114)	0.75	0.454	F	0.358	0.0%
		CC vs CT	2	1.197 (0.816–1.757)	0.92	0.358	F	0.193	41.0%
		CC vs TT	2	0.988 (0.674–1.446)	0.06	0.949	F	0.237	28.4%
		CC + CT vs TT	2	0.855 (0.677–1.081)	1.31	0.191	F	0.756	0.0%
		CT + TT vs CC	2	0.918 (0.835–1.056)	0.47	0.641	F	0.187	42.6%
rs13041247	Overall	C vs T	13	0.874 (0.764–0.999)	1.98	0.048	R	0.000	80.2%
		CC vs CT	13	0.904 (0.733–1.114)	0.98	0.325	R	0.000	69.8%
		CC vs TT	13	0.767 (0.570–1.034)	1.74	0.082	R	0.000	81.4%
		CT vs TT	13	0.839 (0.734–0.959)	2.57	0.010	R	0.0303	50.8%
		CC + CT vs TT	13	0.824 (0.701–0.968)	2.35	0.019	R	0.000	69.7%
		CT + TT vs CC	13	1.185 (0.934–1.503)	1.40	0.162	R	0.000	77.9%

Abbreviations: C vs T, the allele model; CC vs CT, the heterozygote model; CC vs TT, the homozygote model;CC + CT vs TT, the recessive model; CT + TT vs CC, the dominant model; I^2^, I-squared; R, the random effect model; F, the fixed effect model

## Discussion

This meta-analysis incorporated 13 total articles examining the relationship between rs13041247 and NSCL/P risk, with data available for 4914 patients and 5981 controls. In a subgroup analysis of different ethnic groups, we observed no significant association between rs13041247 and NSCL/P risk. However, in an analysis of the overall study population we found the C allele, the CT genotype and the CC + CT model to be significantly linked with reduced NSCL/P risk (C vs T: OR = 0.897, 95% CI: 0.723–1.114, *P* = 0.048; CT vs TT: OR = 0.839, 95% CI: 0.734–0.959, *P* = 0.01; CC + CT vs TT: OR = 0.824, 95% CI: 0.701–0.968, *P* = 0.019). The molecular basis for NSCL/P remains poorly characterized, with both genetic and environmental factors being thought to drive the development of this disease. Risk factors such as maternal drinking, smoking, and poor nutrition prior to conception may influence this risk of this birth defect. Several meta-analyses have recently highlight a number of genetic and environmental risk factors associated with NSCL/P development [19–26].

The MAFB gene is a transcription factor with a basic leucine zipper structure encoded in the 20q12 region [27]. MAFB is a key regulator of the development of endocrine cells, hematopoietic cells, and the development of the orofacial region in addition to functioning in medullary contexts as a tumor suppressor gene [28–30]. Work by Beaty et al. revealed a strong association between NSCL/P risk and SNPs in the MAFB gene in an Asian population. The rs13041247 has been associated with reduced NSCL/P risk in certain studies, whereas others have failed to detect such an association in Caucasian, Brazilian, or Mexican Mestizo populations [7]. A recent meta-analysis by Imani et al. examined the link between the rs13041247 polymorphism and NSCL/P risk [20]. The results of our present analysis were partially consistent with this prior meta-analysis, which had included 10 total studies of Asian, Caucasian, Mixed, and African populations. Our results for the CT vs. TT heterozygote model and the CC + CT vs TT recessive model were similar to those in this previous meta-analysis, whereas our findings regarding the C allele and the CC genotype different from those in this prior study. We found that the C allele was related to NSCL/P risk, whereas the homozygote model was not. This analysis therefore reaffirmed and expanded upon this prior study, expanding the number of included studies to 13, thus yielding a larger sample size that produced some results inconsistent with those of the previous study.

The present meta-analysis has several limitations. For one, the sample size in this analysis was relatively small, limiting our ability to precisely assess the relationship between rs13041247 and NSCL/P risk. Secondly, these analyses were based upon one-way estimates. Furthermore, there was significantly heterogeneity among studies. In addition, we were unable to adjust for maternal risk factors such as alcohol intake or smoking as this information was unavailable in the majority of the included studies. Lastly, we were unable to assess gene-environment and gene-gene interactions for the same reason.

## Conclusion

In summary, the present meta-analysis revealed that the rs13041247 SNP located in the 20q12 region is significantly linked to NSCL/P risk, which the C allele, the CT genotype and the CC + CT model for this SNP being associated with reduced NSCL/P risk in an overall study population. However, subgroup analyses in individual ethnic groups failed to detect any significant relationship between rs13041247 and NSCL/P risk. Future large-scale well-designed studies will therefore be essential to accurately assess the relationship between rs13041247 SNP and NSCL/P risk in order to better understand the etiology of this complex disorder.

## Data Availability

The datasets generated and analyzed during thte current study are available from the corresponding author on reasonable request.

## Abbreviations

C vs T: The allele model
CC vs CT: The heterozygote model
CC vs TT: The homozygote model
CC + CT vs TT: The recessive model
CI: Confidence interval
CL: Cleft lip
CNKI: The China National Knowledge Internet
CP: Cleft palate
CT + TT vs CC: The dominant model
F: The fixed effect model
HWE: Hardy-Weinberg equilibrium
I^2^: I-squared
MAFB: V-maf musculoaponeurotic fibrosarcoma oncogene homolog B
NOS: Newcastle-Ottawa scale
NSCL/P: Nonsyndromic cleft lip with or without cleft palate
OR: Odds ratio
R: The random effect model
SNP: Single nucleotide polymorphisms
